# Bacterial Infection Features in Alcohol-Associated Hepatitis: Review of a 2016–2021 Cohort

**DOI:** 10.3390/jcm13195693

**Published:** 2024-09-25

**Authors:** Cesar Jiménez, Aina Martí-Carretero, Ares Villagrasa, Anna Aguilar, María Pérez-Pérez, Meritxell Ventura-Cots, Victor Vargas

**Affiliations:** 1 Liver Unit, Hospital Universitari Vall d’Hebron, Vall d’Hebron Barcelona Hospital Campus, 08035 Barcelona, Spain; cesar.jimenez@vallhebron.cat (C.J.); meritxell.ventura@vallhebron.cat (M.V.-C.); 2 Department de Medicina, Facultat de Medicina, Universitat Autònoma de Barcelona, 08035 Barcelona, Spain; 3 Vall d’Hebron Research Institute (VHIR), Vall d’Hebron Barcelona Hospital Campus, 08035 Barcelona, Spain; 4 Àrea de Malalties Digestives, Hospital Vall d’Hebron, Vall d’Hebron Barcelona Hospital Campus, 08035 Barcelona, Spain; 5 Centro de Investigación Biomédica en Red Enfermedades Hepáticas y Digestivas (CIBEREHD), Instituto de Salud Carlos III, 28029 Madrid, Spain

**Keywords:** bacterial infections, alcoholic hepatitis, alcohol-associated hepatitis, risk factors, acute-on-chronic liver failure, corticosteroids

## Abstract

**Background/Objectives:** Bacterial infections (BI) are a major cause of mortality in patients with alcohol-associated hepatitis (AH); however, only a few studies have investigated BI in AH in the last decade. Therefore, we aimed to assess the features and outcomes of BI in patients with AH. **Methods:** This observational descriptive study included patients with AH admitted to a tertiary academic hospital between 2016 and 2021. Clinical and complete microbiological data were recorded and complications, including acute-on-chronic liver failure (ACLF), and mortality over 90-days were compared between infected and noninfected patients. **Results:** Overall, 115 patients with AH were recruited and 75 had severe AH; among them, 66 started corticosteroid treatment. We identified 69 cases of BI in 44 patients; the incidence of BI upon hospital discharge was 32.2%, which reached 38.2% at 90 days. The predominant infection site was the chest (35%). Among the identified bacteria (52.1%), half were gram positive and half gram negative. A low rate of multidrug-resistant bacteria (14%) was also noted. Infected patients during hospitalization (*n* = 37) exhibited higher rates of hepatic decompensation and ACLF (*p* = 0.001) and lower survival (81.8% vs. 95.8%, *p* = 0.015) than did noninfected patients (*n* = 78). In-hospital infected patients (n = 22) exhibited worse survival (72.7%) than did those infected upon admission (93.3%) or noninfected patients (94.9%) (*p* = 0.009). Corticosteroid-treated patients displayed a nonsignificant increase in the total number of BI; however, without greater mortality. **Conclusions:** BI were common in our cohort of patients with AH. Patients with in-hospital infections commonly experienced serious complications, including high ACLF and death rates. Infections diagnosed upon admission were treated without affecting survival.

## 1. Introduction

Alcohol-related liver disease (ArLD) is the main cause of chronic liver disease worldwide, contributing to 41.7% of cirrhosis-related deaths [1,2]. It comprises a clinical–histological spectrum, including fatty liver, alcohol-associated hepatitis (AH), and cirrhosis with associated complications. AH is characterized by abrupt jaundice, malaise, and liver-related decompensation [3]. Patients with AH are relatively susceptible to infection [4,5]; some studies have reported up to 49% of infections [4,6]. Both infections and AH potentially lead to acute-on-chronic liver failure (ACLF) with an incidence rate as high as 20–50% at 3 months [7,8].

Studies with the largest cohorts of patients with AH and concomitant infection were conducted more than 10 years ago and all studies did not identify the causative agent [6,9,10,11,12,13]. Moreover, the concept of ACLF was defined in the last decade. Thus, although both AH and infections are the main causes of ACLF, few studies [14,15] have specifically investigated the role of infections in ACLF development in AH.

Therefore, this observational, single-center study endeavored to evaluate the emergence and course of infection in patients with AH. Ultimately, it aimed to describe the characteristics, causative micro-organisms, severity, complications, and risk factors of bacterial infections (BI) in patients with AH.

## 2. Materials and Methods

### 2.1. Study Design and Population

This observational descriptive study included patients with AH consecutively admitted to Hospital Vall d’Hebron liver unit between January 2016 and December 2021 who were followed up for 90 days or until death. Inclusion criteria were age > 18 years, patients with bilirubin level ≥ 3 mg/dL, aspartate aminotransferase (AST) and alanine aminotransferase (ALT) values < 400 IU/L, AST/ALT ratio > 1.5, and active alcohol consumption of >40 g/day in women or >60 g/day in men for ≥6 months with less than 60 days of abstinence prior to inclusion according the AH definition of the National Institute on Alcohol Abuse and Alcoholism (NIAAA) [16] or a confirmatory biopsy. Severe AH was defined as a modified Maddrey discriminant function ≥ 32 upon admission and patients with severe AH were treated with corticosteroids (CS); if they did not exhibit any contraindications, these patients receiving prednisolone were given 40 mg on a daily dose for up to 28 days, according to their response. Nonresponse to CS was defined as a score ≥ 0.45 after 7 days of therapy according to the Lille model. The exclusion criteria were as follows: unclear AH diagnosis; presence of liver disease other than ArLD, such as autoimmune hepatitis, concomitant hepatitis B virus, hepatitis C virus, or human immunodeficiency virus; cocaine use or recent use of a drug with drug-induced liver injury potential within 30 days; prior hematological or solid-organ transplantation; and patients with comorbidities encompassing high short-term mortality including either hepatocellular carcinoma beyond the Milan criteria or extrahepatic neoplasia. The study’s follow-up period ended on day 90 or earlier, on the date of death, or the date of liver transplantation. We compared the characteristics of infected and noninfected patients, whether infections were present upon admission, occurred during hospitalization, or developed over the 90-day follow-up period.

For each patient, the following data were collected upon admission: epidemiological and demographic data such as age, sex, and body mass index; medical records of liver disease, such as diagnosis of hepatic cirrhosis, previous decompensations and previous AH; and other underlying diseases such as arterial hypertension, diabetes mellitus, dyslipidemia, metabolic syndrome, obesity, chronic renal disease, ischemic heart disease, and chronic obstructive pulmonary disease. On admission, infections, hepatic decompensations (HD), acute kidney injury (AKI), ACLF, and severity scores were registered (i.e., Maddrey, Model for End-Stage Liver Disease (MELD), MELD Na, MELD 3.0, and Child–Pugh). Physical, routine laboratory, and microbiological examinations and concurrent medication (prophylactic antibiotic) usage data were also collected. During hospitalization, the following data were recorded: development of infections, HD, AKI, transjugular intrahepatic portosystemic shunt insertion, vasoactive support required, intensive care unit (ICU) support, ACLF development, and mortality. After hospital discharge, only new infections and mortality were recorded. Patients were followed up for 90 days or up to death.

The following information was recorded for infections at any time point: type and location, nosocomial or community acquired infection, type of bacteria, antibiotic resistance and treatment.

### 2.2. Definitions

Hepatic decompensation was defined as the acute development of ascites, upper gastrointestinal bleeding (GIB), hepatic encephalopathy (HE), or any combination of the foregoing, requiring prolonged or new hospitalization [17]. ACLF was defined as a clinical syndrome occurring in patients with cirrhosis characterized by acute deterioration, organ failure, and high short-term mortality, according to the European Foundation for the Study of Chronic Liver Failure criteria [18]. Systemic inflammatory response syndrome (SIRS), sepsis and septic shock were defined according to sepsis criteria [19]. Proven infection (criteria defined by the NACSELD consortium) [4] was established in the following cases: (1) spontaneous bacteremia: positive blood cultures without a source of infection; (2) spontaneous bacterial peritonitis (SBP): ascitic fluid polymorphonuclear cells > 250/µL with or without a positive fluid culture; (3) lower respiratory tract infections: new pulmonary infiltrate on chest radiograph in the presence of compatible clinical criteria, at least one respiratory symptom (cough, sputum production, dyspnea, and/or pleuritic pain), and/or at least one finding on auscultation (rales or crepitation) or sign of infection (e.g., fever and leukocytosis); (4) bacterial enterocolitis: diarrhea or dysentery with a positive stool culture for pathogenic bacteria (e.g., *Salmonella*, *Shigella*, *Yersinia*, *Campylobacter*, and *Escherichia coli*); (5) *Clostridium difficile*: diarrhea with a positive toxigenic *C. difficile* test result; (6) skin infection: cellulitis; (7) urinary tract infection: urinary white blood cell count > 20 per field with positive urinary culture findings in a symptomatic patient; (8) intra-abdominal infections (e.g., diverticulitis, appendicitis, cholangitis, and secondary bacterial peritonitis); and (9) healthcare-associated infections (e.g., catheter-related bloodstream infection (CRBSI)). Multidrug-resistant bacteria (MRB) were defined as nonsusceptibility to at least one agent in at least three antimicrobial categories. Nosocomial infection (in-hospital infection) was defined as “de novo infection” after 72 h of hospitalization.

### 2.3. Statistical Analysis

Descriptive statistics were used to summarize data. Quantitative variables are expressed as the mean ± standard deviation for normally distributed data and as the median (25th–75th percentile) for non-normally distributed data. Percentages were calculated using categorical data. Qualitative variables are presented as frequencies and percentages. Between-group differences for categorical and quantitative variables were evaluated using the chi-square or Fisher’s exact test and the Student’s *t*-test or the Mann–Whitney U test as appropriate. Survival times were compared using the Kaplan–Meier curves and log-rank tests. All statistical analyses were performed using IBM SPSS Statistics (version 22) software.

## 3. Results

### 3.1. Study Population

A total of 169 patients were screened and 115 patients were admitted to our hospital with AH diagnosis and met our eligibility criteria. Their characteristics at baseline are shown in Table 1. In summary, 76.5% were men, the median age was 50 years old, 85% were Caucasians, 70% had alcoholic cirrhosis, more than 50% had HD, and 16% patients were infected.

On admission, 70% of patients were diagnosed with hepatic cirrhosis and 56% exhibited a liver-related decompensation, with ascites being the most common one accounting for up to half of the patients, and 7 (6%) patients fulfilled ACLF criteria. The median (interquartile range) Maddrey, MELD, and Child–Pugh scores were 40 (20–50), 19 (16–22), and 10 (9–11), respectively.

Overall, 75 (65%) patients met the criteria for severe AH (Maddrey score > 32 points); among them, 66 started corticosteroids CS treatment, whereas 9 did not receive CS because of ongoing severe infection and GIB (6 and 3 patients, respectively).

### 3.2. Infections

In the AH cohort, we identified 69 infections in 44 patients (38.2% of all patients with AH) during the 90-day study period. On admission, 20 infections were present in 18 patients (15.6% of all patients with AH).

During hospitalization, 30 infections occurred in 22 patients (19.1% of all patients with AH), among whom 3 had infections upon admission.

**Scheme 1:**
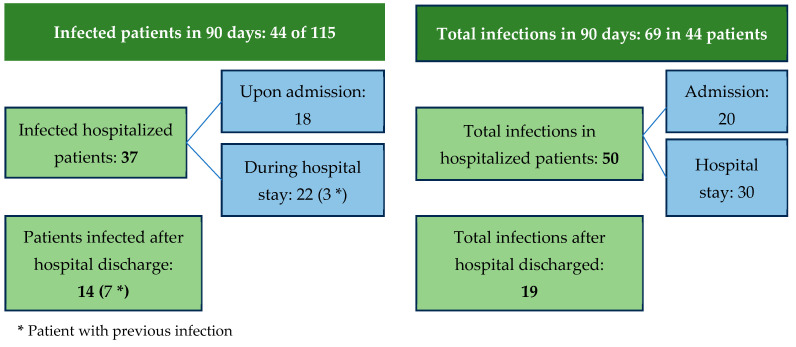
Infected patients and infections at 90 days.

From hospital discharge to the end of follow-up, 19 infections developed in 14 patients (13.2% of 106 living patients), among whom 7 had a previous infection (6 patients had a pre-existing infection upon admission, and 1 developed an infection during hospitalization). The complete information on the 90-day study period is shown in Scheme 1.

### 3.3. Bacteria and Sites of Infection

Among the 69 detected infections, 40 bacteria were identified and isolated from cultures of 36 infections (52.1%). The cultured organisms are listed in Table 2. Overall, gram-positive and gram-negative bacteria were equally represented. Gram-negative bacteria accounted for 50% of the isolated organisms and *E. coli* and *Klebsiella pneumoniae* were the predominantly isolated organisms (17% and 15% of all isolated organisms, respectively). Gram-positive bacteria also accounted for 50% of the isolated organisms and *Staphylococcus aureus* was the most isolated organism (20% of all isolated organisms). MRB were rare in this series of patients; only 5 MRBs (*S. aureus*, *S. haemolyticus*, *K. pneumoniae* (×2) and *Enterococcus faecalis*) were identified among the 36 identified infections (14%).

The infection sites are summarized in Table 2. The chest was the most common infection site, with 24 cases (35%) of pneumonia, followed by the skin (14 (20%)), blood (11 (16%): bacteremia (8) and CRBSI (3)), abdomen (ascites (SBP)) (11 (16%)), and urinary tract (9 (13%)).

### 3.4. Infections upon Admission

On admission, 20 infections were detected in 18 patients, whereas no infection was detected in 97 patients. Two patients had two different infections; one had coexistent SBP, caused by *S. haemolyticus*, and bacteriemia, caused by *Acinetobacter baumannii*, whereas the second had cellulitis and aspiration pneumonia. The most frequently associated infections were chest infections (7 (35%)) and cellulitis (7 (35%)), whereas urinary tract infections (3 (15%)), SBP (2), and bacteremia (1) were the least frequent. In 11 infections (55%), especially pneumonia and cellulitis, the micro-organisms were not identified (clinical diagnosis). Additionally, upon diagnosis, 9 (45%) patients had associated SIRS, whereas 3 (16.6%) met the ACLF criteria. Infection characteristics upon admission and during the course of the disease are shown in Table 3a.

### 3.5. Infections during Follow-Up

A total of 49 infections were detected in these patients (Flowchart), the types of infections chest infections (17 (35%)), blood infections (10 (20%)), and SBP (9 (18%)) were predominantly associated with infections during follow-up. Micro-organisms were identified in a significant proportion of patients (30 out of 49 (61.2%)); half of the isolated organisms were gram-positive bacteria (14 isolations of *Staphylococcus* spp. and 3 of *Enterococcus* spp.), whereas the other half were gram-negative bacteria (mostly *Enterobacteriaceae*: 5 isolations of *E. coli* and 5 of *K. pneumoniae*).

### 3.6. Liver-Related Decompensations and ACLF in Infected Patients Compared with Noninfected Patients

On admission, infected patients (n = 18) yielded higher prognostic scores than did noninfected patients (n = 97). The presence of HD, such as ascites (78% vs. 46%, *p* = 0.029) and HE (50% vs. 6%, *p* = 0.001), was more frequent in infected patients than in noninfected patients. Infected patients exhibited higher prognostic scores than did noninfected patients: Maddrey (48 vs. 38, *p* = 0.02), MELD (22 vs. 19, *p* = 0.01), MELD Na (24.5 vs. 22, *p* = 0.03), MELD 3.0 (25.5 vs. 23, *p* = 0.01), Child–Pugh (11 vs. 10, *p* = 0.001), and ABIC (11 vs. 10; *p* = 0001). Furthermore, infected patients displayed a higher ACLF incidence (16.6% vs. 4.1%, *p* = 0.041) than did noninfected patients. The complete clinical characteristics are shown in Table 4. Notably, infections diagnosed upon admission were cured with antibiotics, thus having no impact on survival.

A comparison of the clinical course of the infected (admission + hospitalization) and noninfected patients (n = 37 vs. n = 78) revealed significant differences. Infected patients (n = 37) developed more cases of HD (62% vs. 28%, *p* = 0.001), especially ascites (43% vs. 23%, *p* = 0.046) and HE (41% vs. 13%, *p* = 0.001), than did noninfected patients. They also presented with ACLF more frequently (32% vs. 6%, *p* = 0.001), requiring vasoactive (16% vs. 1.3%, *p* = 0.002) and ICU (27% vs. 2.5%, *p* = 0.001) support. Finally, patients with infections had a higher mortality rate (6/37, 16% vs. 3/78, 4%; *p* = 0.021) than did noninfected patients. Table 5 compares the clinical characteristics of the infected and noninfected patients. Of the 22 in-hospital infected patients, 10 had infection-associated SIRS, 9 developed ACLF, and 5 died of ACLF (Table 3b). These 22 patients had higher incidence rates of HD (59% vs. 34.4%, *p* = 0.033) and ACLF (36.3% vs. 9.6%, *p* = 0.002) than did the noninfected patients during hospitalization.

We did not identify any significant differences in the incidence rates of infections or liver complications between CS-treated and non-CS-treated patients during hospitalization (Table 6). After discharge, CS-treated patients developed more infections (26% vs. 6%, *p* = 0.09) and had a greater frequency of infection-associated SIRS compared to non-CS-treated patients, but without significant differences. Complete data are provided in Table 3c and Table 6.

### 3.7. Mortality and Predictors of Mortality in Infected Patients

**Figure 1:**
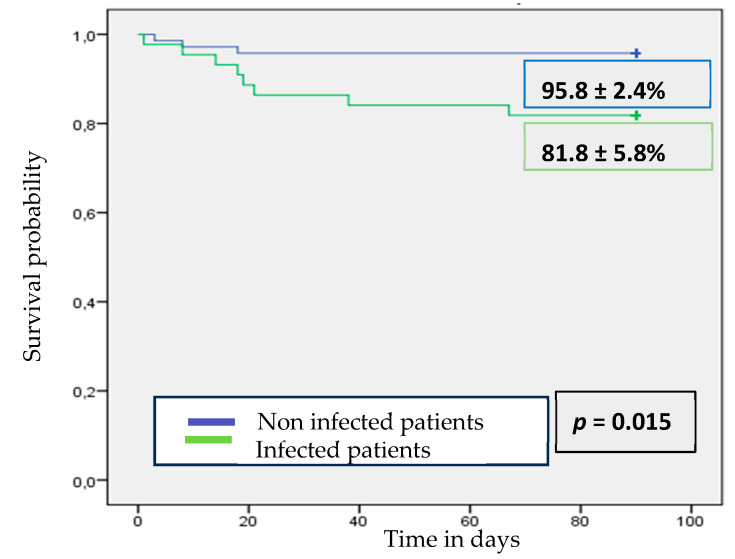
Impact survival according to the infection.

**Figure 2:**
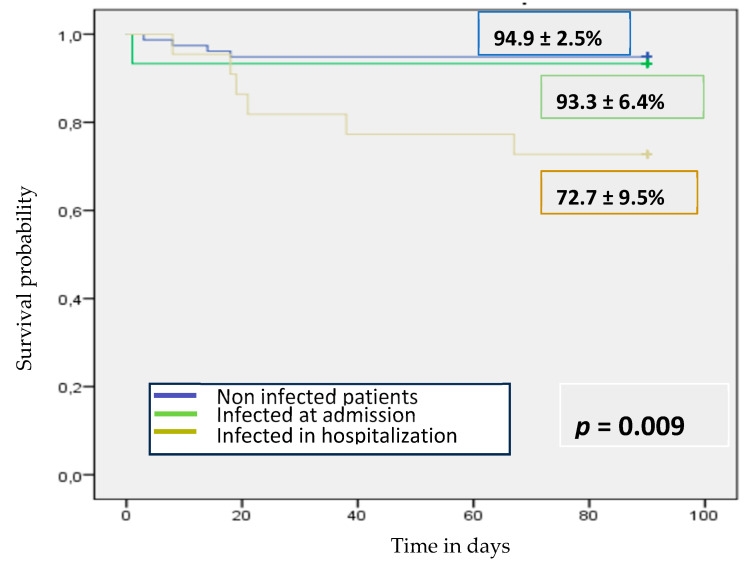
Survival according to the time of the acquisition of infection.

The 90-day survival was higher in noninfected patients (71) than in infected patients (44) (95.8 ± 2.4% vs. 81.8 ± 5.8%, *p* = 0.015; Figure 1). In-hospital infected patients exhibited worse survival (72.7 ± 9.5%) than did those infected upon admission (93.3 ± 6.4%) or noninfected patients (94.9 ± 2.5%) (*p* = 0.009; Figure 2).

## 4. Discussion

In this study, we prospectively reviewed patients who were diagnosed with AH at our academic tertiary hospital and were registered in a national database. Among them, 70% had cirrhosis, 65% had a Maddrey score > 32, and 57% received CS treatment.

In our cohort, we identified 69 infections in 44 patients, among whom the incidence of infection upon hospital discharge was 32.2%, reaching 38.2% at 90 days postdischarge. On admission, 15.6% of patients had infections; however, 19.1% developed in-hospital infections. This incidence was slightly lower than that reported in previous studies. Parker [6] reported a global incidence of 49% for in-hospital infections, whereas Louvet et al. [9] and Michelena et al. [10] reported incidence rates of 25.6% and 23.1%, respectively, among patients infected upon admission, compared to 23% and 43.8%, respectively, among those infected during hospitalization. The incidence of in-hospital infections reported in this study (19.1%) was considerably similar to that observed in a meta-analysis by Hmoud et al. [13], who reported that 20% of CS-treated patients with AH developed in-hospital infections. In contrast, the “Steroids or Pentoxifylline for Alcoholic Hepatitis” study [11] reported that only 10% of patients were infected during hospitalization; however, they accounted for 24% of the deaths in their study.

The predominant infections upon admission were lower respiratory tract infections (7/20) and cellulitis (7/20). Both represent frequent locations of outpatient infections in patients with chronic liver disease. On admission, these infections occurred in patients with more deteriorated liver function; notably, in our series, other common locations, such as the abdomen (ascites) and urinary tract, were underreported. Additionally, in a multicenter study led by Parker [6], patients who acquired in-hospital infections in Spain exhibited relatively few cases of urinary tract infections. In our cohort, infections (pneumonia) were the most frequent in patients with AH who had acquired the infection during hospitalization or follow-up; however, the blood (bacteremia) and abdomen (ascites [SBP]) appeared to be important locations, consistent with other studies [20]. The appearance of these locations is attributable to not only a severe immunological deterioration of patients with AH but also hospitalization (nosocomial infections), prolonged hospital stay, and HD. Ascites and HE predispose to SBP and HE to lower respiratory tract infections (aspiration), respectively. Moreover, secondary mechanical effects on respiratory function owing to abdominal ascites or hydrothorax predispose patients to pulmonary infection [6].

From our infection data, we identified the bacteria responsible for 51% of the infections by culture, displaying consistency with a large multinational study [6] that identified the causative bacteria in 53% of infections. Additionally, we found similar proportions of gram-negative and gram-positive bacteria (50%). Generally, gram-positive bacteria are predominant in skin, chest, and blood infections (catheter-related), whereas gram-negative bacteria are predominant in urinary tract infections and ascites. In our study, the most frequent gram-positive cocci were *S. aureus* and *S. epidermidis*, whereas the predominant gram-negative bacteria were *E. coli* and *K. pneumoniae*. In contrast to a previous study [21] wherein *Enterococcus* was identified as the pathogen, this species was only isolated in three of our patients. Notably, at our center, we found a low incidence of MRB (14%) and only one fungal infection associated with another bacterial infection (CRBSI). This potentially reflects the effectiveness of the antibiotics administered at our center [22,23].

Patients with AH often develop SIRS and immune dysfunction, favoring BI [7,24,25]. Excessive alcohol consumption can induce gut dysbiosis and increase the permeability of the intestinal barrier, inducing bacterial translocation and resulting in endogenous inflammation [26]. Additionally, treatment with CS potentially increases the risk of infection in AH [12,20,27]. Moreover, in a significant proportion of patients with AH, hepatic cirrhosis can also predispose to infection via different mechanisms (immune dysfunction, intestinal dysbiosis, and bacterial translocation) [5,28]. Both situations possibly explain the presence of infections in AH (38% of patients in our study). In our study, BI resulted in a poor prognosis for patients with AH. Infected patients had worse survival rates and more instances of HD, especially ascites and HE, compared to noninfected patients. In addition, infection favored ACLF development in patients with AH. ACLF is a syndrome characterized by acutely decompensated cirrhosis, associated with single or multiple organ failure with a high risk of short-term death (i.e., death < 28 days after hospital admission) [29]. ACLF occurs in the context of intense systemic inflammation and BI are one of the key factors; in this sense, bacterial translocation is associated with high levels of circulating pathogen-associated molecular patterns (PAMPs) such as lipopolysaccharide (LPS) and gram-negative endotoxins, which are major Toll-like receptor 4 (TLR4) ligands expressed in injury liver tissue, and its activation is responsible for inducing exacerbated inflammation [30]. In the context of intense systemic inflammation, it frequently develops in close temporal relationships with proinflammatory precipitating events and is associated with single or multiple organ failure [31]. Among the most frequent proinflammatory precipitation events in Europe are infection and AH [32]. In our study, the coexistence of infection with AH induced a high rate (32%) of ACLF with a severe course of ICU admission in several cases. Considering the prognostic implications of ACLF, this is an important finding since most previous studies have not considered the presence of ACLF because the syndrome had not been defined or used yet.

CS treatment in patients with AH seems to increase susceptibility to infection [9,11,12,20]; nevertheless, CS use is safe once the infection is under control. A meta-analysis of 12 randomized trials reported a 12% cumulative incidence of infection in patients with AH during CS therapy [13]. Although the treatment groups (CS and non-CS) in our study were not comparable, CS-treated and non-CS-treated patients presented no differences in the incidence of infections or mortality rate. This is consistent with the findings of Hmoud et al. [13], who found that CS did not increase BI-related mortality in patients with severe AH.

Similar to other studies, we categorized infections based on onset time and peri- and postadmission diagnoses (in-hospital and follow-up infections). However, our data on follow-up infections were limited to those available in the registry; therefore, they were not included in the analysis. Nonetheless, infections upon admission in our study had two remarkable characteristics. First, on admission, infected patients had more advanced liver disease than did noninfected patients, based on the HD, Child–Pugh, and MELD scores. According to the PREDICT [31] and other studies on cirrhosis, infection and liver impairment are followed by greater susceptibility to HD and ACLF. This characteristic was not observed in previous studies, such as by Louvet et al. [9]. Second, the infection upon admission exhibited favorable evolution. In our cohort, all infections upon admission resolved with adequate antibiotic treatment and no deaths were recorded. In these patients, infection cure, outcome, and survival were similar to those in noninfected patients. However, not all series have made similar observations; in a multicenter study, Parker et al. [6] did not identify any differences in survival between infected patients upon admission and those infected during hospitalization.

Consistent with the study by Michelena et al. [10], one of the most important findings in our study was that patients infected during hospitalization presented a greater number of complications and yielded a higher mortality rate than did noninfected patients. The reason for this poor evolution is that infections develop concurrently with proinflammation, leading to immune paralysis and predisposing to severe infections [25]. Additionally, CS use and infections by MRB potentially contribute to infection severity; in our cohort, we observed 2 MRB (*Enterobacter cloacae*, *Klebsiella pneumoniae*) infections linked to ACLF and death. ACLF was the clinical complication that conferred the poorest prognosis to these patients; in fact, out of 22 in-hospital infected patients, 9 (41%) developed ACLF and 5 died. The fact that in-hospital infections have the worst prognostic value suggests the possibility of considering the use of antibiotic prophylaxis upon admission in patients with severe AH receiving CE. [14,33,34,35].

Despite the positive results, this study has some limitations. First, although we collected data on all clinical events, the biochemical data recorded during hospitalization were only collected at baseline. Second, we lacked information after hospital discharge as only two postdischarge clinical events were recorded: infections and death. Thus, no biochemical information was available to evaluate prognostic scores or other liver-related complications. Finally, we did not obtain information on alcohol consumption after discharge.

## 5. Conclusions

Infections were common in a cohort of patients with AH admitted to a tertiary academic hospital. The most frequent site of infection was the chest. Among the identified bacteria, half of the isolated organisms were gram positive and the other half were gram negative. The number of infections caused by enteric bacteria predominantly gram negative (in abdomen: ascites, UTI) was evenly equaled among chest, blood, and skin infections, with gram-positive bacteria predominating in these sites. Infections diagnosed upon admission were cured with antibiotics and had no impact on survival. Our findings suggest that in-hospital infections are commonly associated with serious complications in patients with AH, including a high rate of ACLF and death. Corticosteroid-treated patients displayed a nonsignificant increase in the total number of infections and this was not accompanied by greater mortality.

## Figures and Tables

**Table 1:** Baseline clinical and biological characteristics.

	n = 115
Hospital Admission (BASELINE)	
**Sex (male), n (%)**	88 (76.5%)
**Age, median (P 25–75)**	50 (44–58)
**Race, n (%)**	Caucasian 98 (85%)
**BMI, median (P 25–75)**	27 (24–31)
**Hepatic cirrhosis, n (%)**	81 (70%)
**Hepatic decompensation, n (%)**	64 (56%)
**Infection, n (%)**	18 (16%)
**Ascites, n (%)**	59 (51%)
**Hepatic encephalopathy, n (%)**	15 (13%)
**Gastrointestinal bleeding, n (%)**	7 (6%)
**Acute kidney injury, n (%)**	13 (11%)
**Acute-on-chronic liver failure, n (%)**	7 (6%)
**Maddrey score, median (P 25–75)**	40 (20–50)
**MELD score, median (P 25–75)**	19 (16–22)
**MELD Na score, median (P 25–75)**	22 (19–22)
**MELD 3.0 score, median (P 25–75)**	23 (20–26)
**Child–Pugh score, median (P 25–75)**	10 (9–11)
**Bilirubin (mg/dL), median (P 25–75)**	7.4 (4.8–12)
**INR, median (P 25–75)**	1.5 (1.2–1.8)
**Albumin (g/dL), median (P 25–75)**	2.7 (2.4–3.1)
**Creatinine (mg/dL), median (P 25–75)**	0.7 (0.5–0.9)
**AST (UI/L), median (P 25–75)**	147 (102–264)
**ALT (UI/L), median (P 25–75)**	61 (35–89)
**GGT (UI/L), median (P 25–75)**	593 (223–1461)
**ALP (UI/L), median (P 25–75)**	204 (150–328)
**CRP (mg/dL), median (P 25–75)**	2.5 (1–5.5)
**Leucocytes (10^9^** **/L), median (P 25–75)**	8.2 (6.1–11.6)
**Platelets 10^9^/L (P 25–75)**	104 (64–153)

ALT: alanine aminotransferase, ALP: alkaline phosphatase, AST: aspartate transaminase, BMI: body mass index, CRP: C-reactive protein, GGT: gamma-glutamyl transferase, INR: international normalized ratio, MELD: model for end-stage liver disease, P 25–75: 25th–75th percentile.

**Table 2:** Site infection and bacteria classification (gram stain).

Site	Number of Infections, (%)	Positive Cultures	Bacteria, (n)	Gram Positive or Gram Negative, (n)
**Chest**	24 (35%)	3	*Streptoccocus pneumoniae*, *Staphylococcus aureus*, *Clamydia pneumoniae*	Gram positive (2) gram negative (1)
**Skin**	14 (20%)	3	*Staphylococcus aureus* (2) Staphylococcus aureus *	Gram positive (3)
**Blood**	11 (16%)	11	*Acinetobacter baumannii Staphylococcus hemolyticus* (2) Staphylococcus hemolyticus * Staphylococcus epidermidis (4) Enterococcus faecalis *, *Klebsiella oxytoca*, *Staphylococcus aureus*, *Escherichia coli*, *Klebsiella pneumoniae*, *Enterococcus faecium*	Gram positive (10) gram negative (4)
**Abdominal (Ascites)**	11 (16%)	10	*Staphylococcus aureus* (2) *Acinetobacter baumannii Staphylococcus hemolyticus Escherichia coli*, *Enterobacter cloacae*, Klebsiella pneumoniae * Enterococcus faecium, *Serratia marcenses*, *Acinetobacter pittii*	Gram positive (4) gram negative (6)
**Urinary tract**	9 (13%)	9	*Escherichia coli* (5), *Klebsiella pneumoniae* (2), Klebsiella pneumoniae * Enterococcus faecalis, *Klebsiella aerogenes*	Gram positive (1) gram negative (9)
**TOTAL**	69	36	40	Gram positive (20) gram negative (20)

* Multidrug-resistant bacteria.

**Table 3:** (**a**) Characteristics of bacterial infections at admission. (**b**) Characteristics of infections in hospitalization. (**c**) Characteristics of infections after hospital discharge.

(a)										
ID-Episode	Infection		Bacteria	SIRS	ACLF	Resolution Infection	Antibiotics			MRB
**P4-1**	SBP		*Staphylococcus aureus*	Yes	No	Yes	Cefazolin			
**P6-2**	Cellulitis		Nonisolated bacteria	Yes	No	Yes	Levofloxacin + Clindamycin			
**P8-3**	Aspiration Pneumonia		Nonisolated bacteria	Yes	No	Yes	Amoxicillin-Clavulanate			
**P17-4**	Cellulitis		Nonisolated bacteria	No	No	Yes	Amoxicillin-Clavulanate			
**P19-5**	Pneumonia		Nonisolated bacteria	Yes	No	Yes	Amoxicillin-Clavulanate			
**P33-6**	Bacteremia		*Acinetobacter baumannii* + *Staphylococcus haemolyticus*	Yes	No	Yes	Ciprofloxacin			
	SBP									
**P62-7**	Cellulitis		Nonisolated bacteria	No	No	Yes	Amoxicillin-Clavulanate			
**P69-8**	Cellulitis		Nonisolated bacteria	Yes	No	Yes	Amoxicillin-Clavulanate			
**P74-9**	UTI		*Escherichia coli*	No	No	Yes	Ceftriaxone			
**P76-10**	Pneumonia		*Streptococcus pneumoniae*	Yes	Yes	Yes	Cefotaxime + Azithromycin			
**P79-11**	Pneumonia		*Chlamydia pneumoniae*	No	No	Yes	Levofloxacin			
**P82-12**	UTI		*Klebsiella pneumoniae*	Yes	Yes	Yes	Meropenem			
**P86-13**	Pneumonia		Nonisolated bacteria	No	No	Yes	Amoxicillin-Clavulanate			
**P26-14**	Aspiration Pneumonia		Nonisolated bacteria	No	No	Yes	Piperacillin-Tazobactam			
**P27-15**	Cellulitis		Staphylococcus aureus	No	No	Yes	Amoxicillin-Clavulanate			
**P89-16**	Aspiration Pneumonia		Nonisolated bacteria	Yes	Yes	Yes	Piperacillin-Tazobactam			
	Cellulitis		Nonisolated bacteria							
**P99-17**	Cellulitis		Nonisolated bacteria	No	No	Yes	Amoxicillin-Clavulanate			
**P107-18**	UTI		*Escherichia coli* + *Enterococcus faecalis*	No	No	Yes	Amoxicillin-Clavulanate			
**(b)**										
**ID-Episode**	**CE**	**Infection**	**Bacteria**	**SIRS**	**ACLF**	**Resolution Infection**	**Cause of Death**	**Antibiotic**		**MRB**
**P2-1**	Yes	CRBSI	*Staphylococcus epidermidis*	Yes	Yes	Yes	ACLF	Meropenem		
**P4-2**	Yes	UTI	*Klebsiella aerogenes*	Yes	Yes	Yes		Cefazolin		
		Cellulitis	*Staphylococcus aureus*					Cloxacillin		
		CRBSI	*Enterococcus faecalis*, *Staphylococcus epidermidis*, *Staphylococcus haemolyticus*					Piperacillin-Tazobactam		
**P5-3**	Yes	UTI	*Escherichia coli*	No	No	Yes		Ceftriaxone		
**P6-4**	Yes	Aspiration Pneumonia	Nonisolated bacteria	No	No	Yes		Meropenem		
**P20-5**	No	UTI	*Escherichia coli*	No	No	Yes		Amoxicillin-Clavulanate		
**P21-6**	Yes	Aspiration Pneumonia	Nonisolated bacteria	Yes	Yes	Yes	ACLF	Ceftazidime		
**P28-7**	Yes	Pneumonia	Nonisolated bacteria	No	No	Yes		Amoxicillin-Clavulanate		
**P38-8**	No	Cellulitis	*Staphylococcus aureus*	No	No	Yes		Linezolid		Yes
**P44-9**	No	Pneumonia	Nonisolated bacteria	No	No	Yes		Amoxicillin-Clavulanate		
**P50-10**	Yes	Bacteremia	*Klebsiella oxytoca*	Yes	No	Yes		Amoxicillin-Clavulanate		
**P51-11**	Yes	Pneumonia	Nonisolated bacteria	No	No	Yes		Piperacillin-Tazobactam		
**P25-12**	No	SBP	*Staphylococcus aureus*	Yes	Yes	Yes		Ceftriaxone		
		Bacteremia	*Staphylococcus aureus*					Cefazolin		
		Aspiration Pneumonia	Nonisolated bacteria					Piperacillin-Tazobactam		
**P61-13**	Yes	Pneumonia	Nonisolated bacteria	Yes	Yes	Yes	ACLF	Piperacillin-Tazobactam		
**P63-14**	No	SBP	*Escherichia coli*	No	No	Yes		Ceftriaxone		
		Pneumonia	Nonisolated bacteria							
**P68-15**	Yes	Bacteriemia	*Escherichia coli*	Yes	Yes	Yes		Piperacillin-Tazobactam		
**P73-16**	No	Aspiration Pneumonia	Nonisolated bacteria	No	No	Yes		Amoxicillin-Clavulanate		
**P78-17**	Yes	SBP	*Enterobacter cloacae*	Yes	Yes	No	SBP, ACLF	Ceftriaxone		
		Aspiration Pneumonia	Nonisolated bacteria					Amoxicillin-Clavulanate		
		SBP	*Klebsiella pneumoniae*					Meropenem + Daptomycin		Yes
**P82-18**	No	Pneumonia	Nonisolated bacteria	Yes	Yes	No	ACLF	Meropenem		
		CRBSI	*Staphylococcus hemolyticus*, *Staphylococcus epidermidis*. Candida albicans *					Meropenem + Daptomycin, Anidulafungin *		Yes
**P87-19**	No	Aspiration Pneumonia	Nonisolated bacteria	No	No	Yes		Amoxicillin-Clavulanate		
**P102-20**	No	Pneumonia	Nonisolated bacteria	No	No	Yes		Ceftriaxone		
**P103-21**	Yes	Pneumonia	Nonisolated bacteria	Yes	Yes	Yes		Piperacillin-Tazobactam		
**P106-22**	No	Aspiration Pneumonia	Nonisolated bacteria	No	No	Yes		Piperacillin-Tazobactam		
**(c)**										
**ID-Episode**	**CE**	**Infection**	**Bacteria**	**SIRS**	**ACLF**	**Resolution Infection**	**Cause of Death**		**Antibiotic**	**MRB**
**P5-1**	Yes	Bacteremia	*Staphylococcus epidermidis*	No	No	Yes			Ceftriaxone	
**P19-2**	Yes	Cellulitis	Nonisolated bacteria	No	No	Yes			Ceftriaxone, Teicoplanin	
**P28-3**	Yes	Pneumonia	*Staphylococcus aureus*	Yes	Yes	No	Septic Shock		Meropenem + Linezolid	
		Intra-abdominal	Nonisolated bacteria							
**P33-4**	Yes	SBP	*Enterococcus faecium*	Yes	No	Yes			Piperacillin-Tazobactam	
**P39-5**	Yes	Cellulitis	Nonisolated bacteria	No	No	Yes			Amoxicillin-Clavulanate	
**P45-6**	Yes	Bacteriemia	*Klebsiella pneumoniae*	Yes	Yes	Yes			Meropenem, Teicoplanin	
		Bacteremia	*Enterococcus faecium*							Yes
**P47-7**	Yes	SBP	*Serratia marcescens*	No	No	Yes			Ceftriaxone	
**P26-8**	No	Bacteremia	*Staphylococcus aureus*	No	No	Yes			Amoxicillin-Clavulanate	
**P74-9**	Yes	UTI	*Escherichia coli*	No	No	Yes			Ceftriaxone	
**P76-10**	No	Cellulitis	Nonisolated bacteria	No	No	Yes			Cefadroxil	
**P83-11**	No	Cellulitis	Nonisolated bacteria	No	No	Yes			Amoxicillin-Clavulanate	
**P92-12**	Yes	SBP	*Klebsiella pneumoniae*	No	No	Yes			Ceftriaxone	
		UTI	*Klebsiella pneumoniae*	No	No	Yes			Ciprofloxacin	
		UTI	*Klebsiella pneumoniae*	Yes	Yes	yes			Cefotaxime + Clindamycin	Yes
		Cellulitis	Nonisolated bacteria	No	No	Yes			Amoxicillin-Clavulanate	
**P105-13**	Yes	Pneumonia	Nonisolated bacteria	No	No	Yes			Piperacillin-Tazobactam	
**P108-14**	Yes	SBP	*Acinetobacter pitti*	Yes	Yes	Yes	ACLF		Meropenem	

ACLF: acute-on-chronic liver failure, CE: corticosteroids, CRBSI: catheter-related bloodstream infection, MRB: multidrug-resistant bacteria, SBP: spontaneous bacterial peritonitis, UTI: urinary tract infection. * Anidilafingin is treatment for *Candida albicans*.

**Table 4:** Clinical and biological characteristics at admission.

	Infected Patients n = 18	Noninfected Patients n= 97	*p*
**Sex (male), n (%)**	12 (66.6%)	75 (77.3%)	0.28
**Age, median (P 25–75)**	45 (41.7–55.7)	51 (44–58)	0.43
**BMI, median (P 25–7527)**	27.3 (23–31.2)	27.2 (24–31.7)	0.86
**Hepatic cirrhosis, n (%)**	16 (88%)	65 (67%)	0.062
**Hepatic decompensations, n (%)**	16 (88%)	48 (49.5%)	**0.005**
**Ascites, n (%)**	14 (78%)	45 (46.4%)	**0.029**
**HE, n (%)**	9 (50%)	6 (6.2%)	**0.001**
**GIB, n (%)**	0	7 (7.2%)	0.25
**AKI, n (%)**	3 (16.6%)	10 (10.3%)	0.43
**ACLF, n (%)**	3 (16.6%)	4 (4.1%)	**0.041**
**Maddrey score, median (P 25–75)**	48 (32–68.5)	38 (18–51)	**0.022**
**MELD score, median (P 25–75)**	22 (19–25)	19 (15–21)	**0.012**
**MELD Na score, median (P 25–75)**	24 (22–29)	22 (19–26)	**0.037**
**MELD 3.0 score, median (P 25–75)**	25 (25–28)	23 (20–25)	**0.013**
**Child–Pugh score, median (P 25–75)**	11 (11–12)	10 (9–11)	**0.011**
**Bilirubin (mg/dL), median (P 25–75)**	9.89 (6.96–16.31)	7.1 (4.5–11.6)	0.084
**INR, median (P 25–75)**	1.8 (1.4–1.95)	1.5 (1.15–1.8)	**0.027**
**Creatinine (mg/dL), median (P 25–75)**	0.63 (0.55–0.87)	0.67 (0.54–0.88)	0.81
**Albumin (g/dL), median (P25–P75)**	2.5 (2.3–2.7)	2.8 (2.4–3.2)	**0.038**
**CRP (mg/dL), median (P 25–75)**	3 (1.5–9.5)	2.4 (0.9–5.2)	0.31
**Leucocytes (10^9^/L), median (P 25–75)**	8.2 (5.8–10.7)	10.3 (6.6–13.2)	0.26

AKI: acute kidney injury, ACLF: acute-on-chronic liver failure, BMI: body mass index, CRP: C-reactive protein, GIB: gastrointestinal bleeding, HE: hepatic encephalopathy, INR: international normalized ratio, MELD: model for end-stage liver disease, P 25–75: 25th–75th percentile.

**Table 5:** Baseline and complications, patients in hospitalization.

	Infected Patients n = 37	No Infected Patients n = 78	*p*
**Sex (male), n (%)**	25 (67.5%)	63 (81%)	0.12
**Age, median (P 25–75)**	50 (43–57)	51 (44–58)	0.87
**Race, n (%)**	Caucasian, 30 (87%)	Caucasian, 68 (87%)	0.91
**BASELINE**			
**Maddrey score, median (P 25–75)**	45 (31.5–61.5)	37 (18–51)	**0.020**
**MELD score, median (P 25–75)**	21 (18–25)	18 (15–21)	**0.002**
**Child–Pugh score, median (P 25–75)**	11 (10–12)	10 (9–11)	**0.001**
**Bilirubin (mg/dL), median (P 25–75)**	10.4 (5.4–17.5)	6.5 (4.4–10.5)	**0.010**
**INR, median (P 25–75)**	1.63 (1.4–1.84)	1.48 (1.14–1.80)	**0.047**
**Albumin (g/dL), median (P25–P75)**	2.5 (2.3–2.75)	2.8 (2.5–3.4)	**0.002**
**Creatinine (mg/dL), median (P 25–75)**	0.7 (0.6–1.0)	0.65 (0.53–0.86)	0.45
**COMPLICATIONS**			
**Hepatic decompensations, n (%)**	23 (62%)	22 (28%)	**0.001**
**Ascites, n (%)**	16 (43%)	18 (23%)	**0.046**
**HE, n (%)**	15 (41%)	10 (13%)	**0.001**
**GIB, n (%)**	4 (11%)	5(6.4%)	0.41
**AKI, n (%)**	6 (16%)	5 (6.4%)	0.095
**Vasoactive support**	6 (16%)	1 (1.3%)	**0.002**
**ICU, n (%)**	10 (27%)	2 (2.5%)	**0.001**
**ACLF, n (%)**	12 (32.4%)	5 (6.4%)	**0.001**
**Death, n (%)**	6 (16%)	3 (4%)	**0.021**

ACLF: acute-on-chronic liver failure, AKI: acute kidney injury, GIB: gastrointestinal bleeding, HE: hepatic encephalopathy, ICU: intensive care unit, INR: international normalized ratio, MELD: model for end-stage liver disease, P 25–75: 25th–75th percentile.

**Table 6:** Clinical characteristics, noncorticosteroid treatment vs. treated patients.

	No Corticosteroids n = 49	Corticosteroids n = 66	*p*
**BASELINE**			
**Male, n (%)**	36 (73.5%)	51 (77%)	0.63
**Age, median (P 25–75)**	51 (44–58)	48 (43–56)	0.16
**Maddrey score, median (P 25–75)**	34 (18–50)	41 (28–58)	0.27
**MELD score, median (P 25–75)**	19 (15–22)	19 (16–22)	0.88
**Child–Pugh score, median (P 25–75)**	10 (9–11)	10 (9–11)	0.44
**COMPLICATIONS**			
**Infections, n (%)**	9 (18%)	13 (20%)	0.76
**Hepatic decompensations, n (%)**	18 (36%)	27 (40%)	0.65
**Ascites, n (%)**	14 (28%)	20 (30%)	0.89
**HE, n (%)**	10 (20%)	15 (22%)	0.73
**GIB, n (%)**	2 (4%)	7 (10%)	0.19
**AKI, n (%)**	7 (14%)	4 (6%)	0.14
**Vasoactive support, n (%)**	3 (6%)	4 (6%)	0.98
**ICU, n (%)**	5 (10%)	7 (11%)	0.94
**ACLF, n (%)**	4 (8%)	13 (20%)	0.08
**Death, n (%)**	4 (8%)	5 (7.5%)	0.92
**Follow up 90 days**			
**Infections, n (%)**	3/45 (6%)	11/61 (18%)	0.08
**Number of infections, n (%)**	3/45 (6%)	16/61 (26%)	0.09
**Death, n (%)**	1 (2%)	1 (1.5%)	0.82

ACLF: acute-on-chronic liver failure, AKI: acute kidney injury, GIB: gastrointestinal bleeding, HE: hepatic encephalopathy, ICU: intensive care unit, MELD: model for end-stage liver disease, P 25–75: 25th–75th percentile.

## Data Availability

The raw data supporting the conclusions of this article will be made available by the authors on request.
